# Prevalence, clinical profile, maternal and perinatal outcomes of pregnancies complicated with obstructed labor at a teaching hospital in Tigray, Ethiopia: A five-year retrospective cross-sectional study

**DOI:** 10.1371/journal.pone.0328007

**Published:** 2025-07-11

**Authors:** Hale Teka, Mussie Alemayehu, Awol Yemane, Marta Abrha, Tsega Gebremariam, Ephrem Berhe, Bisrat Tesfay Abera, Ashenafi Tekle, Habtom Tadesse, Hiluf Ebuy Abraha, Yibrah Berhe

**Affiliations:** 1 Department of Obstetrics and Gynecology, School of Medicine, College of Health Sciences, Mekelle University, Mekelle, Ethiopia; 2 Department of Reproductive Health, School of Public Health, College of Health Sciences, Mekelle University, Mekelle, Ethiopia; 3 Department of Internal Medicine, School of Medicine College of Health Sciences, Mekelle University, Mekelle, Ethiopia; 4 Quality Assurance Office, School of Medicine, College of Health Sciences, Mekelle University, Mekelle, Ethiopia; 5 Arnold School of Public Health, University of South Carolina, Columbia, South Carolina, United States of America; University of the Witwatersrand, SOUTH AFRICA

## Abstract

**Background:**

Obstructed labor remains a significant direct obstetric complication that leads to increased maternal and neonatal morbidity and mortality, particularly in resource-poor settings. Understanding the prevalence, clinical profile and maternal and perinatal outcomes is critical to developing targeted interventions to improve maternal and neonatal survival.

**Methods:**

An institutional based retrospective cross-sectional study was conducted at Ayder Comprehensive Specialized Hospital (ACSH) in Tigray, Ethiopia, between 2017 and 2021. Sociodemographic, obstetric and clinical data as well as maternal and neonatal outcomes were extracted and analysed using descriptive statistics.

**Results:**

The prevalence of obstructed labor during the study period was 0.38% [95% CI: 0.36% to 0.42%]. The mean age of the mothers was 30 years (SD = 6.04), half were between 25 and 34 years old. Most mothers (89%) were from the Tigray region, while the remaining were from neighbouring districts of Afar and Amhara regional states. More than two-thirds of these mothers lived in rural areas. Eighty-two percent attended at least one antenatal care visit, and 87% were referred. The average duration of labor was 14.5 hours [IQR = 8.24]. On admission, 28% were hypotensive, 65% were tachypneic, and nearly 68% had no fetal cardiac activity. Maternal complications included uterine rupture (65%), anemia (78%), postpartum hemorrhage (71%), and sepsis (23%). There were two maternal deaths. Cephalopelvic disproportion was present in 73% of cases. There were dismal neonatal outcomes with 70% mortality.

**Conclusions:**

Obstructed labor continues to be a life-threatening obstetric emergency in this region, resulting in severe maternal complications, maternal deaths and extremely high neonatal mortality rates. Strengthening referral systems, improving capacity for emergency obstetric care, raising public awareness and early interventions are essential to reduce these avoidable burdens and achieve maternal and neonatal health goals.

## Introduction

Globally, maternal mortality remains a pressing issue, with an estimated 287,000 maternal deaths in 2020 [1]. Although the global maternal mortality ratio (MMR) fell from 385 to 223 per 100,000 live births between 1990 and 2020, the improvements are uneven [1,2]. Women in low-income countries, particularly in sub-Saharan Africa, bear a disproportionate burden and are responsible for more than 60% of maternal deaths [1,3]. Limited access to skilled birth attendance, trained personnel and robust referral systems contribute significantly to this disparity [3,4]. In contrast, high-income countries that benefit from timely access to quality obstetric care experience far fewer maternal deaths [2,5]. Strengthening health infrastructures and ensuring rapid, skilled emergency obstetric care can prevent most maternal deaths [4,6].

Obstructed labor is one of the main causes of maternal mortality in resource-poor settings, arising when the fetal presenting part fails to descend despite adequate contractions [7,8]. The term obstructed labor denotes a failure to progress due to mechanical problems such as mismatch between fetal size (size of the presenting part, pathologically enlarged fetal head), malpresentation, dysfunctional labor and tumor previa obstructing the lower uterine segment (7,8). The resulting prolonged pressure leads to ischemia, necrosis and devastating complications such as uterine rupture, postpartum hemorrhage, sepsis, fistula formation, stillbirth and neonatal death [8,9]. Globally, obstructed labor is responsible for about 8% of maternal deaths [7,10]. In Ethiopia, although maternal mortality rates have improved, obstructed labor remains a critical problem due to late management, weak referral systems, and limited emergency obstetric services [11–14]. Addressing these gaps—through better referral pathways, improved surgical capacity, increased antenatal care and community education—could reduce maternal and neonatal mortality [6,10,15].

The aim of this study was to investigate the, prevalence, clinical profile and maternal and perinatal outcomes of mothers with obstructed labor in a tertiary referral hospital in Tigray, Ethiopia. By elucidating the prevalence, severity, and complications, the results can inform strategic interventions to strengthen emergency obstetric care, thereby advancing Ethiopia’s maternal health goals and aligning with global targets to ensure safe childbirth [1,6,10].

## Methods

### Study area, study period, and study design

An institutional based retrospective cross-sectional study was conducted at Ayder Comprehensive Specialized Hospital (ACSH), one of the largest tertiary hospitals in the Tigray region of Ethiopia, between 2017–2021. ACSH hosts an average of 5000 deliveries per year. It utilises the Modified WHO partograph (2000) and heavily relies on intermittent auscultation for fetal monitoring and palpation for counting uterine contractions.

### Study population and sample size

All cases of obstructed labor from 2017 to 2021 were included, defined as failure of the fetal presenting part to descend despite adequate contractions. This study was conducted among 83 women diagnosed with obstructed labor and consecutively admitted to ACSH from 2017 to 2021.

### Study variables and source of data

Data were extracted using EpiData v4.6 with a standardized checklist capturing sociodemographic variables, obstetric history, clinical parameters, maternal complications, neonatal outcomes, causes of obstructed labor, and interventions. Maternal mortality and neonatal mortality (defined here as zero APGAR at five minutes) were noted. Data were analyzed descriptively. Patient anonymity was ensured throughout the process by removing identifiers.

### Data collection procedure

Data were collected by trained healthcare professionals. EpiData version 4.6 was used as data entry software to maintain skip logic, data consistency, typing error, and ease data export to statistical software. Cases were identified via labor ward logbooks and operating room records.

### Data analysis

Data were exported to STATA 16 for analysis. Categorical variables are described using frequency, percent with its 95% confidence interval, and graphs. Continuous variables are described using a relevant combination of measure of central tendency and measure of dispersion.

### Ethical considerations

Ethical approval was obtained from the Institutional Review Board (IRB) (MU-IRB 1950/2022) of Mekelle University, College of Health Sciences. This was part of a large maternal near-miss and mortality research project conducted in ACSH. Permission for data collection was obtained from the Chief Clinical Director (CCD) of ACSH. Informed consent was not required as we used secondary data. Patient charts were accessed and reviewed from May 1, 2022 – June 30, 2022.

## Results

**Fig 1:**
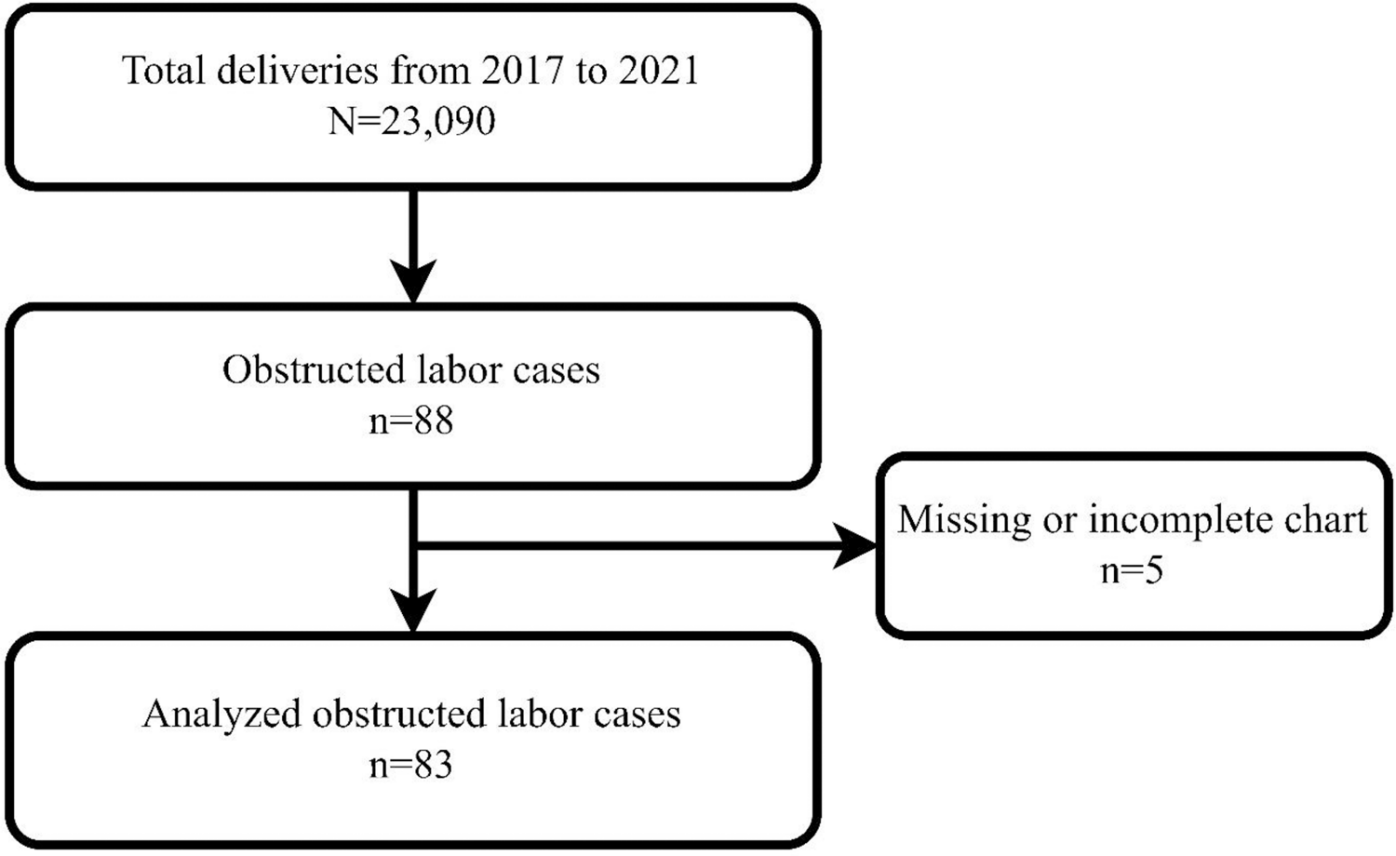
Flow chart of total deliveries and obstructed labor cases in ACSH from 2017 to 2021.

Between 2017 and 2021 Ayder Comprehensive Specialized Hospital recorded 23 090 deliveries; 88 (0.38%, 95% CI 0.36–0.42%) fulfilled the diagnostic criteria for obstructed labour. Five charts were incomplete, leaving 83 cases for analysis (Fig 1).

### Sociodemographic characteristics and obstetric profile of women with obstructed labor

The women’s mean age was 30 ± 6 years (range 15–45); one-half were 25–34 years old and almost two-thirds were rural residents (Table 1). Majority of women (89.19%) were from Tigray, while the remaining mothers were from neighbouring districts of Afar and Amhara regional states.

**Table 1:** Sociodemographic characteristics and obstetric profile of women with obstructed labor at Ayder Comprehensive Specialized Hospital (ACSH), Tigray, Ethiopia, 2017–2021 (n = 83).

Variables	n (%)	95% CI
**Region**		
Tigray	74(89.16)	80.2,94.3
Others (Afar and Amhara)	9(10.84)	5.68,19.7
**Residence**		
Rural	54(65.1)	54.0,74.6
Urban	29(34.9)	25.35,45.9
**Age**		
20-24	16(19.28)	12,07, 29.34
25-34	42(50.6)	39.8,61.3
35-45	25(30.1)	21.1, 40.9
**Gravida**		
One	9(10.84)	5.68-19.70
2-4	44(53.01)	42.1-63.60
5 and above	30(36.14)	26.4-47.10
**Parity**		
Zero	10(12.0)	6.64-21.10
One	12(14.46)	8.32-23.90
2-4	40(48.19)	37.5-59.00
5 and above	21(25.3)	17.0-35.80
**Number of SVD**		
Zero	11(13.25)	7.4-22.50
One	16(19.28)	12.07-29.34
Two	21(25.30)	17.01-35.80
3 and above	35(42.17)	31.9-53.10
**Number of CS**		
Zero	60(72.29)	61.5-80.90
One	23(27.71	19.05-38.43
**History of Abortion**		
Yes	13(15.6)	9.24-25.20
No	70(84.34)	74.7-90.75
**ANC visits**		
Yes	68(81.93)	71.99-88.8
No	15(18.07)	11.10-28.0
**Means of arrival**		
Referred	72(86.75)	77.40-92.50
Self	11(13.25)	7.42-22.50
**Place of diagnosis**		
On referral	42(50.60)	39.8-61.30
In ACSH	41(49.40)	38.67-60.10
**Labor duration (n = 36)**		
12 hours and less	21(61.76)	44.0-76.80
>12 hours	13(38.24)	23.1-55.90
**Median duration of ROM**	10 [6,16] hours	

Median gravidity and parity were four and three, respectively. Nearly 82% had attended at least one antenatal care visit. While 72 (86.75%) of the mothers came through referral, only 42 (58.33%) of these were referred with the diagnosis of obstructed labor. This might stand for two things: either the referring healthcare professionals failed to pick up obstructed labor or the mothers took too long to arrive at our center and they developed obstructed labor. The median duration of labor and rupture of membrane were 14.5 h (IQR 8–24), and 10 h (IQR 6–16), respectively.

On admission, 28% were hypotensive, 65% tachypnoeic, and 15% febrile; fetal heart tones were absent in 68% of cases (Table 2). Anaemia (Hb < 11 g/dL) affected 55% and thrombocytopenia (< 100 × 10^9/L) was present in 13%. Signs of advanced obstruction were common—abdominal tenderness (67%), severe moulding (defined as skull bones overlapping and cannot be easily separated with pressure) (45.78%), caput (significant scalp edema preventing an appropriate appreciation of the suture lines and fetal head position (47%), vaginal bleeding (39%) and haematuria (25%). Two maternal deaths yielded a case-fatality rate of 2.4% (Table 2).

**Table 2:** Condition on arrival of women with obstructed labor at Ayder Comprehensive Specialized Hospital (ACSH), Tigray, Ethiopia, 2017–2021 (n = 83).

Variables	n (%)	95% CI
**Hypotensive**		
Yes	23(27.71)	19.05-38.4
No	60(72.29)	61.5-80.9
**Tachypnea**		
Yes	54(65.06)	54.03-74.64
No	29(34.94)	25.3-45.9
**Fever (n = 33)**		
Yes	5(15.15)	6.2-32.43
No	28(84.85)	67.56-93.77
**Fetal heartbeat**		
Present	26(32.1)	22.7-43.1
Absent(zero)	55(67.9)	56.8-77.25
**Platelet count**		
<100,000	8(12.5)	6.29-23.3
100,000+	56(87.5)	76.6-93.7
**Hemoglobin (n = 73)**		
<11mg/dl	40(54.79)	43.13-65.9
>=11 mg/dl	33(45.21)	34.0-56.88
**Three tumor abdomen**		
Yes	14(16.87)	10.17-26.6
No	69(83.13)	73.34-9.83
**Easily palpable fetal part**		
Yes	37(44.58)	34.14-55.52
No	46(55.42)	44.48-65.86
**Tender abdomen**		
Yes	56(67.47)	56.55-76.77
No	27(32.53)	23.23-43.45
**Vaginal bleeding**		
Yes	32(38.55)	28.6-49.56
No	51(61.45)	50.44-71.4
**Vulvar edema**		
Yes	23(27.71)	19.05-38.44
No	60(72.29)	61.56-80.95
**Sever molding**		
Yes	38(45.78)	35.27-56.69
No	45(54.22)	43.31-64.73
**Excessive caput**		
Yes	39(46.99)	36.4-57.86
No	44(53.01)	42.14-63.6
**Hematuria**		
Yes	21(25.30)	17.01-35.89
No	62(74.70)	64.11-82.99
**Distended bowel**		
Yes	7(4.03-16.81)	4.03-16.81
No	76(91.57)	83.19-95.67
**Edematous bladder**		
Yes	51(61.45)	50.44-71.4
No	32(38.45)	28.6-49.56
**Maternal Death**		
Yes	2 (2.40%)	0.3–8.5
No	81 (97.60%)	91.6–99.7

### Maternal complications, cause, and management among mothers with obstructed labor

Uterine rupture occurred in 54 (65%) of women, postpartum haemorrhage in 71%, and sepsis in 23%. Moreover 9.6% developed obstetric fistula. Anaemia necessitated transfusion in 70%. Iatrogenic visceral injury complicated 6% of surgeries. Hospital stays exceeded seven days for 53% of patients. Cephalopelvic disproportion (CPD) a condition diagnosed in our setting when there is either excessive caput or severe moulding in the face of labor progress abnormalities was the most common cause of obstructed labor accounting for nearly two-third of the cases (Table 3).

**Table 3:** Maternal complication and cause for obstructed labor among women with obstructed labor at Ayder Comprehensive Specialized Hospital (ACSH), Tigray, Ethiopia, 2017–2021 (n = 83).

Variables	n(%)	95% CI
**Anemia**		
Yes	65(78.31)	68.0,85.9
No	18(21.69)	14.0,31.99
**Uterine rupture**		
Yes	54(65.06)	54.0,74.6
No	29(34.94)	25.3,45.9
**Sepsis**		
Yes	19(22.89)	15.0,33.2
No	64(77.11)	66.7,84.9
**PPH**		
Yes	59(71.08)	60.2,79.9
No	24(28.92)	20.0.39.7
**Obstetric fistula**		
Yes	8(9.64)	4.84,18.27
No	75(90.36)	81.7,95.1
**Wound dehiscence**		
Yes	3(3.7)	1.1,11.0
No	78(96.3)	88.9,98.8
**Blood transfusion**		
Yes	49(70)	58.1,79.69
No	21(30)	20,3,41.89
**Hysterectomy**		
Yes	42(50.6)	39.8,61.3
No	41(49.4)	38.67,60.17
**Relaparatomy**		
Yes	11(13.25)	7.42,22.5
No	72(86.75)	77.4,92.5
**Iatrogenic organ injury**		
Yes	5(6.1)	2.5,13.99
No	78(93.9)	86.0,97.4
**Cause of OL**		
CPD	61(73.31)	61.6,80.9
Malpresentation	11(13.25)	7.42,22.53
Malposition	3(3.61)	1.11,10.7
Contracted pelvis	2(2.41)	0.59,9.3
Fetal congenital anomaly	1(1.2)	0.01,827
Unknown	5(6.02)	2.61,24.9
**Anti-acid given**		
Yes	1(1.2)	0.01,8.27
No	82(98.8)	91.7,98.35
**Hospital stays**		
<= 7-days	39(46.9)	36.9,57.85
>7 days	44(53.01)	42.14,63.6

CPD – Cephalopelvic disproportion; PPH – Postpartum hemorrhage; OL – Obstructed labor.

### Neonatal outcomes for mothers with obstructed labor

Neonatal outcomes were poor. Apgar scores were zero at both one and five minutes in 57 infants (70%), and only 20% achieved an Apgar ≥ 7 at five minutes. Low birth weight (< 2 500 g) was documented in 11% of newborns; 6% weighed ≥ 4 000 g. Male infants accounted for 55% of births, and sex was unrecorded in 17% of charts (Table 4).

**Table 4:** Neonatal outcome of women with obstructed labor at Ayder Comprehensive Specialized Hospital (ACSH), Tigray, Ethiopia, 2017–2021 (n = 83).

Variables	n(%)	95% CI
**APGAR score at first minute**		
0	57(70.37)	59.4,79.4
1-6	14(17.28)	10.4,27.2
7 and above	10(12.35)	6.7,21.6
**APGAR score at 5**^**th**^ **minute**		
0	57(70.37)	59.4,79.4
1-6	8(9.88)	4.96,18.69
7 and above	16(19.75)	12.37,30.0
**Weight of the neonate**		
Less than 2500 gram	7(10.77)	5.14,21.1
2500 to 3999 grams	54(83.08)	71.7,90.48
≥ 4000 grams	4(6.15)	2.28,15.5
**Sex of the neonate**		
Male	38(55.07)	18.1,39.4
Female	19(27.54)	43.06,66.5
Undocumented	12(17.39)	10.0.28.4

## Discussion

This study aimed to investigate the prevalence, clinical profile, and maternal and neonatal morbidities and mortalities associated with obstructed labor at Ayder Comprehensive Specialized Hospital, 2017–2021. Out of 23,090 deliveries during the study period, 88 were diagnosed with Obstructed Labor giving the prevalence of 0.38% (95% CI: 0.36%, 0.42%), i.e., 3.8 obstructed labor per 1000 deliveries. The leading cause of obstructed labor was CPD. Obstructed labor resulted in life-threatening complicaations such as uterine rupture, hemorrhage, anemia, and sepsis. In the present study there were two maternal deaths (2.41%, 95% CI, 0.3% to 8.5%.). In this study, 70% neonatal mortality was recorded. These findings underscore the urgent need for timely detection, referral and comprehensive emergency obstetric care to prevent avoidable maternal and neonatal deaths.

Although the prevalence in the present study is lower than some reports of 1–5% in other resource-poor areas [16–18], it still represents a significant risk given the severity of complications. In high-income regions, the prevalence is usually much lower thanks to effective surveillance and timely surgical interventions [5,9]. The relatively moderate prevalence in this study could be due to slightly better referral patterns or population characteristics compared to more remote or resource-limited areas, although it is still higher than the negligible rates in advanced health systems.

In this study, uterine rupture occurred in 65% of cases, a figure that is significantly higher than the 5–20% reported in other resource-poor countries [7,8,10,16]. For example, a study from southern Nigeria found a uterine rupture rate of about 10–15% in obstructed labor [17], while rates in some South Asian studies are often below 20% [18]. The high rate in our setting suggests that women are being admitted at an advanced stage of obstruction due to systemic delays at all levels—community detection, referral and facility response. Strengthening the capacity of the health system to recognize and treat obstructed labor early could significantly reduce the incidence of uterine rupture.

Anemia (78%) and postpartum hemorrhage (71%) were also more common than in reports from other regions. Studies in Tanzania and Malawi reported anemia and PPH in about 40–60% of stillbirth cases [19,20], lower than our findings. The high rates of anemia could be due to chronic nutritional deficiencies exacerbated by acute blood loss, while severe hemorrhage indicates delayed interventions. Expanding blood banking services, ensuring iron supplementation and prompt surgical treatment are needed to improve outcomes.

Sepsis (23%) and obstetric fistula (10%) were further serious complications. In other resource-poor countries, sepsis rates are around 10–20% [7,15], somewhat lower than in our study, and fistula rates vary widely but are generally lower when timely intervention is possible [21,22]. The increased prevalence of these morbidities suggests prolonged obstructed labor, repeated examinations, and suboptimal infection control measures. Targeted improvements in aseptic techniques, antibiotic prophylaxis and timely operative delivery would likely mitigate such infections and prevent debilitating outcomes such as fistula formation.

The maternal mortality in our study—two deaths in 83 women—is remarkable, considering that both maternal deaths and near misses reflect weaknesses in the health care system [23]. Other studies from referral hospitals in low-income settings report lower maternal mortality rates in obstructed labor due to earlier interventions [7,10,18]. Our findings emphasize that rapid detection, improved referral pathways, adequately skilled staff and appropriate surgical capacity can reduce maternal deaths. Ensuring ambulance services, well-trained staff and the availability of comprehensive emergency obstetric care must remain a priority.

Neonatal mortality rate of 70% showcases the catastrophic nature of obstructed labor. While in some resource-poor countries the perinatal mortality rate for obstructed labor is 30–50% [10,16,24], our figure is significantly higher. This indicates ongoing fetal distress and missed opportunities for timely delivery by cesarean section or assisted operative delivery. Improved fetal monitoring, rapid decision making for surgical intervention and well-equipped neonatal resuscitation teams could significantly reduce these tragic losses.

The findings presented here are consistent with several regional studies that emphasize that obstructed labor is a major cause of maternal and neonatal mortality [10,11,14,25]. They emphasize the importance of integrating obstetric care into primary health care systems, strengthening referral networks and ensuring facility readiness. Educating the community about the danger signs of labor, timely ANC visits, and birth preparedness can help women seek care earlier and reduce the severity of obstructions on arrival.

## Strengths and limitations

This five-year retrospective analysis in a tertiary center captures severe, and complex casesof obstructed labor. Although comprehensive, the retrospective design may have introduced bias in data collection, and the focus on one institution limits generalizability. Future multicenter, prospective studies are needed to strengthen external validity and investigate causal factors. The root causes for the delayed presentation, factors contributing to the neglect, and possible solutions to mitigate the systemic barriers should be assessed.

## Conclusions

Obstructed labor continues to be a serious obstetric emergency in this tertiary referral hospital, resulting in severe maternal complications, maternal deaths, and an exceptionally high neonatal mortality rate. It is imperative to address the systemic delays through improved referral pathways, skilled emergency obstetric care, blood transfusion services, robust infection prevention measures and increased community sensitization. In this way, Ethiopia could move closer to national and global maternal and child health targets and ensure that preventable maternal and neonatal deaths from obstructed labor are drastically reduced.

## Supporting information

S1 File Obstructed labor. (XLS)
